# The Role of Inflammation and Infection in Age-Related Neurodegenerative Diseases: Lessons From Bacterial Meningitis Applied to Alzheimer Disease and Age-Related Macular Degeneration

**DOI:** 10.3389/fncel.2021.635486

**Published:** 2021-03-26

**Authors:** Lay Khoon Too, Nicholas Hunt, Matthew P. Simunovic

**Affiliations:** ^1^Faculty of Medicine and Health, Save Sight Institute, The University of Sydney, Sydney, NSW, Australia; ^2^Discipline of Pathology, Faculty of Medicine and Health, The University of Sydney, Sydney, NSW, Australia; ^3^Sydney Eye Hospital, Sydney, NSW, Australia

**Keywords:** infection, neuroinflammation, neurodegenerative disease, Alzheimer disease, bacterial meningitis, age-related macular degeneration

## Abstract

Age-related neurodegenerative diseases, such as Alzheimer disease (AD) and age-related macular degeneration (AMD), are multifactorial and have diverse genetic and environmental risk factors. Despite the complex nature of the diseases, there is long-standing, and growing, evidence linking microbial infection to the development of AD dementia, which we summarize in this article. Also, we highlight emerging research findings that support a role for parainfection in the pathophysiology of AMD, a disease of the neurosensory retina that has been shown to share risk factors and pathological features with AD. Acute neurological infections, such as Bacterial Meningitis (BM), trigger inflammatory events that permanently change how the brain functions, leading to lasting cognitive impairment. Neuroinflammation likewise is a known pathological event that occurs in the early stages of chronic age-related neurodegenerative diseases AD and AMD and might be triggered as a parainfectious event. To date, at least 16 microbial pathogens have been linked to the development of AD; on the other hand, investigation of a microbe-AMD relationship is in its infancy. This mini-review article provides a synthesis of existing evidence indicating a contribution of parainfection in the aetiology of AD and of emerging findings that support a similar process in AMD. Subsequently, it describes the major immunopathological mechanisms that are common to BM and AD/AMD. Together, this evidence leads to our proposal that both AD and AMD may have an infectious aetiology that operates through a dysregulated inflammatory response, leading to deleterious outcomes. Last, it draws fresh insights from the existing literature about potential therapeutic options for BM that might alleviate neurological disruption associated with infections, and which could, by extension, be explored in the context of AD and AMD.

## Introduction

Tissue inflammation, which was described initially as an outcome of the host defense mechanism against intruding pathogens and injury, is now also considered a hallmark of aging, due to “inflammageing”. This term was coined (Franceschi et al., 2000) to describe the decline in immune function due to aging and the immunological shift towards a pro-inflammatory profile. It remains unclear whether the inflammation is a driver of (“pathogenic”), or a response to (“protective”), a degenerative condition. In infection-associated neuroinflammation such as occurs in bacterial meningitis (BM) the inflammatory process which comprises multiple networks of protein mediators and cellular players acts as a double-edged sword: it eliminates intruding pathogens but simultaneously causes bystander immune pathology.

Although the brain and the eye are considered to be immunologically privileged by dint of the blood-brain and blood-retina barriers respectively neuroinflammation has been detected in both organs. Immune contributions to Alzheimer disease (AD; Hensley, 2010) and age-related macular degeneration (AMD; Buschini et al., 2011) are now also recognised. But what are the triggers for “inflammageing”? In the context of AD, the “plaque” theory has postulated that the neuropathological hallmarks of AD [amyloid plaques, neurofibrillary tangles (NFT), and neurodegeneration] are neuroinflammatory triggers (Hensley, 2010). In AMD, lipoproteins and free radicals are known initiators of retinal parainflammation, a form of chronic low-grade inflammation caused by endogenous stress (Xu et al., 2009). Furthermore, several genes in the complement system, such as *complement factor H (CFH)*—whose product regulates complement-mediated inflammation—are associated with AMD (Geerlings et al., 2017). Parainfectious triggers of neurodegeneration, i.e., infection in the central nervous system (CNS) or systemic infection, have recently attracted significant research attention. This has been supported by histopathological, epidemiological, and microbiome findings (Itzhaki et al., 2020; Komaroff, 2020). Regardless of the identity of such triggers, it is believed that they might not only initiate an immune response, but that they also potentiate such responses through persistence.

This mini literature review begins by providing a succinct overview of the role of infections in AD, focussing on the latest developments without extensively covering material that has been reviewed elsewhere by other authors (Fulop et al., 2018; Moir et al., 2018; Morris et al., 2018; Trempe and Lewis, 2018; Ashraf et al., 2019; Moir and Tanzi, 2019; Komaroff, 2020). Since, AD and AMD share common risk factors, e.g., aging and smoking, and pathologic features, e.g., the presence of extracellular deposits and complement system activation (Kaarniranta et al., 2011), this mini-review article also integrates recent evidence that suggests a contribution of parainfection in the aetiology of AMD. It then focuses on the immunopathological properties common to the two age-related neurodegenerative diseases and BM—a neuroinfectious disease that often leads to lifelong neurological disorders. Extensive research effort on BM has been put into alleviating its associated lifelong neurological disabilities *via* adjunct immunomodulation. We aim to provide original insights that may bridge the research gap between a neurological infectious disease, BM, and age-related neurodegenerative diseases, AD and AMD, for which there is increasing evidence of an infectious aetiology.

## Existing Evidence Suggests An Infectious Aetiology of Neurodegenerative Diseases

**Figure 1:**
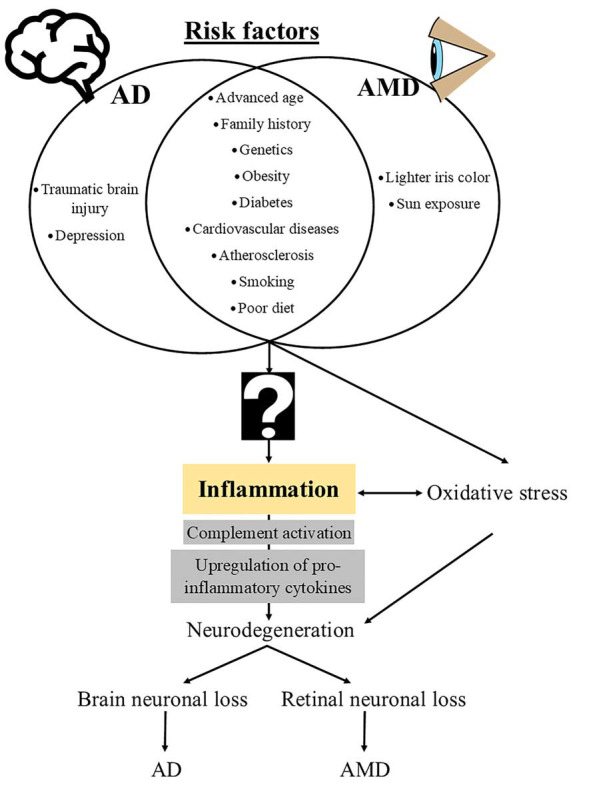
Schematic diagram of risk factors and mechanistic events that underlie the pathogenesis of Alzheimer disease (AD) and age-related macular degeneration (AMD). The question sign indicates an unknown, potentially “infectious” contributor to the downstream immunopathological cascades that are common in both AD and AMD.

The neurodegenerative diseases AD and AMD are complex multifactorial diseases that share modifiable (e.g., treatable medical conditions and lifestyle factors) and non-modifiable (e.g., age and genetics) risk factors as well as common pathological mechanisms, including inflammation and oxidative stress (Figure 1).

### Alzheimer Disease

AD, an irreversible, progressive neurodegenerative disorder, contributes to 60%–80% of dementia cases among the elderly (Alzheimer’s Association, 2015). It is a complex multifactorial disease that has a strong genetic component with more than 50 risk loci identified (Silva et al., 2019). Mutations in the genes *APP, PSEN1*, and *PSEN2* encoding for amyloid precursor protein, presenilin 1 and presenilin 2, respectively, account for most of the early-onset AD, while mutated apolipoprotein E (*APOE*) gene is frequently associated with late-onset AD (Silva et al., 2019). Such genetic defects were initially linked to amyloidosis—a well-known histopathological hallmark of AD, the removal of which has been attempted as a treatment; unfortunately, such clinical trials have failed (Oxford et al., 2020). While this appears to preclude amyloidosis as a primary causal factor for AD, it does not diminish the importance of genetic factors in AD, and continued efforts to give biological meaning to genetic information may facilitate the identification of aetiological agent(s) and/or key immunopathological factor(s), which ultimately are necessary for therapeutic discovery.

#### Theories of AD Aetiology

There are two principal theories of AD aetiology. The first, and earliest, is amyloid-β (Aβ)—centric: it proposes a causal relationship between Aβ and AD. The second, alternative, theory proposes that Aβ does not directly cause AD, given that the known neuropathological hallmarks—accumulation of amyloid plaques around brain neurons and the formation of NFT—are also present in other neurodegenerative diseases, including post-stroke syndrome (Thiel et al., 2014), traumatic brain injury (Kenney et al., 2018) and lead poisoning (Li et al., 2010). Moreover, neuroimaging and post-mortem histopathology show the presence of Aβ deposits and NFT in cognitively normal elderly people (Fagan et al., 2009; Price et al., 2009; Chetelat et al., 2013). One extension of this latter hypothesis is that the plaques and tangles seen in AD represent a stereotypical response to inflammation, which in turn is initiated by an infectious agent. This is backed by several lines of evidence, including detection of microbial components in AD biospecimens and empirical demonstration of the antimicrobial properties of Aβ, which is consistent with its production as part of a host defense mechanism to eliminate infectious agents (Soscia et al., 2010). Numerous reviews are proposing the alternative theory of microbiological pathogenesis in AD, with overlapping and distinct perspectives (Fulop et al., 2018; Moir et al., 2018; Morris et al., 2018; Trempe and Lewis, 2018; Ashraf et al., 2019; Moir and Tanzi, 2019; Komaroff, 2020). Itzhaki and colleagues, and Komaroff, recently enunciated their viewpoints on the infectious aetiology of AD (Itzhaki et al., 2020; Komaroff, 2020). In general, scientific evidence for an infectious aetiology of AD comes from histopathological, epidemiological, and molecular findings.

There is a long-standing thesis of herpes simplex virus type 1 (HSV1) as a risk factor for AD among apolipoprotein E gene (APOE-ε4) carriers (Itzhaki, 2018). It proposes the latent presence of the virus in the human brain, with limited reactivation beyond middle age triggering chronic neuroinflammation that eventually escalates into progressive neurodegeneration (Itzhaki et al., 1997; Itzhaki, 2018). Likewise, infection by the human immunodeficiency virus (HIV) remains a significant aetiological factor in HIV-associated dementia, which reproduces the defining hallmarks of AD (Fischer-Smith and Rappaport, 2005). Other AD-associated neurotropic viruses include HSV6, *Cytomegalovirus* (CMV), *Epstein*–*Barr virus*, *Varicella*–*Zoster virus*, and *Hepatitis C virus* (Sochocka et al., 2017). On the other hand, several spirochetal infections have been implicated in AD development and progression (Miklossy, 2015). To date, epidemiological and neuropathological studies have identified at least 16 microbial pathogens, including seven bacteria (such as *Chlamydia pneumoniae*) as having a role in the development of AD (Sochocka et al., 2017; Balin et al., 2018; Ashraf et al., 2019). Fungal contagion by *Candida albicans* (Pisa et al., 2015) and parasitic infection by *Toxoplasma gondii* (Nayeri Chegeni et al., 2019) also have been linked to AD. In the 2000s, two cases of reversible AD were reported in which patients pre-diagnosed with AD recovered from neurological and cognitive symptoms following antifungal treatment for cryptococcal meningitis (Ala et al., 2004; Hoffmann et al., 2009).

### Age-Related Macular Degeneration

AMD is clinically defined as progressive loss of central vision resulting from neuronal and non-neuronal degeneration in the macula, the central part of the retina that is responsible for the finest spatial, temporal, and spectral acuity. The mainstay of management is secondary preventative treatments: in particular, there is a widespread use of anti-vascular endothelial growth factor agents (anti-VEGF) to manage disease progression for neovascular “wet” AMD, but there is no currently approved therapy for the atrophic “dry” form of AMD. There are multiple risk factors for AMD, including advanced age, genetic polymorphisms, light iris color, smoking, and a high-fat diet (Lambert et al., 2016). The first two are the two strongest risk factors, with genetic polymorphisms of *CFH* and *age-related maculopathy susceptibility 2 (ARMS2)* accounting for more than 50% of the heritability of AMD (DeAngelis et al., 2017). Although both AD and AMD have a strong genetic component, none of the AD-associated genes are linked to AMD pathology, and* vice versa* (Kaarniranta et al., 2011). Given the complex multifactorial nature of AMD, the precise aetiological sequence remains elusive.

The concept of an infectious aetiology of AMD largely stems from direct evidence of a serological association between microbes and AMD, indirect evidence that links microbe-associated diseases to AMD, or evidence that implicates microbe-mediated inflammatory responses to AMD pathogenesis. Three case-control studies separately established a significant serological association between wet and/or dry AMD and *C. pneumoniae* infection (Ishida et al., 2003; Kalayoglu et al., 2003; Shen et al., 2009), an infectious pathogen that has emerged as a risk factor for common non-infectious diseases, including AD and cardiovascular disease. AMD patients with high antibody titers of *C. pneumoniae* were also found to have a 2- to 3-fold increased risk of disease progression (Robman et al., 2005). Another case-control study, however, found a significant association between CMV infection and both forms of AMD, but not infection with *C. pneumoniae* and *H. pylori* (Miller et al., 2004), while four other studies reported no significant association between AMD and *C. pneumoniae* or *Mycoplasma pneumoniae* (Klein et al., 2005; Haas et al., 2009; Turgut et al., 2010; Khandhadia et al., 2012). The inconsistent findings might have been attributable to variations of types and stages of AMD cases included in the studies (Chen et al., 2014), or underlying variations in other factors contributing to the aetiology of AMD.

While activation of the complement system is central to controlling microbial infection, it also is a well-recognised player in AMD pathogenesis. This is demonstrated by the identification of complement signalling protein constituents in drusen—a pathological hallmark of AMD, the accumulation of which disrupts retinal homeostasis supported by retinal pigment epithelium (RPE; Anderson et al., 2010; Kawa et al., 2014; Weber et al., 2014; McHarg et al., 2015). Second, genetic polymorphisms of complement pathway-inhibiting genes contribute to an increased risk of AMD (Lambert et al., 2016). Of particular interest, the membrane cofactor protein CD46, which was found to be downregulated in the RPE of early geographic AMD, is an HHV-6A-specific receptor (Vogt et al., 2011). In multiple sclerosis, HHV-6A infection in astrocytes leads to CD46 downregulation, resulting in hyperactivation of the complement system that is damaging to the local tissue (Pinter et al., 2000). It, therefore, has been speculated that HHV-6A infection may trigger the pathological events that eventually lead to AMD development and progression (Fierz, 2017).

## Immunopathological Mechanisms in Bm That Are Shared with Age-Related Neurodegenerative Diseases

BM is frequently caused by *Streptococcus pneumoniae* and *Neisseria meningitidis*, with the former being associated with high mortality rate or lifelong neurological sequelae in patients with good recovery (Brouwer et al., 2010; also refer to review Liechti et al., 2015 for the pathophysiology of BM). Although there is no direct serological evidence that links primary infectious pathogens of BM to AD or AMD, these diseases share several common inflammatory events that may disrupt normal physiological processes. Similar to other infectious diseases, CNS infection by BM microbes typically triggers an inflammatory response that comprises four elements: the inducers, sensors, mediators, and effectors (Medzhitov, 2010). While the “inducers” of AD/AMD can be multifactorial and of infectious origin not directly linked to BM, the “sensors,” “mediators” and “effectors” overlap between BM and AD/AMD (Table 1). The discovery of inflammation-related sensors, mediators, and effectors in age-related diseases, e.g., AD and AMD (Eikelenboom et al., 2000; Franceschi et al., 2000; Buschini et al., 2011), leads to the current understanding that inflammation is not exclusively a response to tissue injury or infectious diseases. It also occurs throughout life, triggered by various endogenous or exogenous factors, and becomes pathological as a result of immunosenescence (Ferrucci and Fabbri, 2018). Although it begins at different levels of intensity (acute and heightened for BM vs. low and sustained during aging (Franceschi et al., 2017), inflammation initially acts to protect the host by eliminating invading pathogens (BM) or endogenous waste (aging). However, in both cases, inflammation becomes dysregulated by the yet-to-be-identified pathological factor(s), causing a cascade of deleterious immunological events that subsequently damage local neurons, eventually leading to functional loss at the inflammatory loci.

**Table 1:** Key players in innate immunity that underlie the pathogenesis of bacterial meningitis, and potentially Alzheimer disease and age-related macular degeneration.

Immunological players		Bacterial meningitis	Alzheimer disease	Age-related macular degeneration
Sensors	Complement system	Classic, lectin and **alternative** complement pathways	Classic and **alternative** complement pathways	**Alternative** complement pathways
	Other PRRs	TLR1, **TLR2**, **TLR4**, TLR5, TLR6, TLR9, **CD14**, NOD1, NOD2, **NLRP3**	**TLR2**, **TLR4**, **CD14**, NLRC4, NLRP1, **NLRP3**	**TLR2**, TLR3, **TLR4**, TLR9, **CD14**, CD36, NOD1, NOD2, **NLRP3**, RAGE
Mediators	Cytokines	TNF, IL-6, **IL-1β**, IFN-γ, IL-10, TGF-β	TNF, IL-6, **IL-1β**, IFN-γ, TGF-β	IL-18, **IL-1β**
	Chemokines	CCL1, **CCL2**, CCL3-4, CCL8, CCL9, CCL11-12 CCL15, CCL18, CCL20, CCL24-25, CXCL1-2, CXCL4-5, CXCL7, CXCL8, CXCL10, CXCL12-13, CXCL16, MIF, XCL-1	**CCL2**, CCL5, CXCL8, CXCL10	**CCL2**
Effectors	Immune cells	Neutrophils (predominant), **monocytes**, **microglia**, astrocytes, **macrophages**, **T cells**, endothelial cells, ependymal cells	**Microglia**, **macrophages**, astrocytes, **monocytes**, neutrophils, **T cells**	**Microglia**, **macrophages**, **monocytes**, dendritic cells, **T cells**, retinal pigment epithelia, choroidal endothelial cells
References		Nockher et al. (1999), Polfliet et al. (2001), Koedel (2009), Braun et al. (2011), Mook-Kanamori et al. (2011), Coutinho et al. (2013), Geldhoff et al. (2013), Mamik and Power (2017), and Thorsdottir et al. (2019)	Landreth and Reed-Geaghan (2009), Domingues et al. (2017), Jevtic et al. (2017), Kong et al. (2017), Mamik and Power (2017), and Krance et al. (2019)	Kaarniranta and Salminen (2009), Ambati et al. (2013), Camelo (2014), and Chen et al. (2015)

*Shared properties are bolded. Abbreviations: CCL, CC chemokine ligand; CD, cluster of differentiation; CXCL, CXC chemokine ligand; IFN-γ, interferon-gamma; IL, interleukin; MIF, macrophage migration inhibitory factor; NOD, Nucleotide oligomerisation domain; NLRP, nucleotide-binding domain, leucine-rich—containing family, pyrin domain—containing; PRR, pattern recognition receptor; RAGE, receptor for advanced glycation end products; TGF-β, transforming growth factor-beta; TLR, toll-like receptor; TNF, *tumour necrosis factor; XCL-1,* chemokine (C motif) ligand*.

### Complement Pathways

During BM, the first line of innate immune defense involves activation of the host classical complement pathway to label the invading pathogen for eradication by immune cells, with subsequent inhibition of the alternative complement pathway by complement receptor 1, CFH, and complement protease complement factor I to prevent an excessive, tissue-damaging immune response. Activation of the classical complement pathway is induced by C-reactive protein (CRP), upregulation of which is common to BM (Prasad et al., 2005) and both AD (O’Bryant et al., 2010) and AMD (Molins et al., 2018). Furthermore, genetic polymorphism of the non-coding *CFH* gene has been shown to associate with reduced CFH level in the cerebrospinal fluid and increased mortality in both clinical and experimental BM (Kasanmoentalib et al., 2019). This major allele (G) rs6677604 has also been described as a risk factor for AMD (Ansari et al., 2013). While there is no genetic linkage of this allele to AD, another risk allele, rs1061170, is common to both AD and AMD (Zhang et al., 2016), and molecular analyses of drusen (AMD) and senile plaques (AD) reveal the presence of common complement components, including C3, C5, C6-9, and factors B, H, and I (Sivak, 2013), implying the occurrence of a common inflammatory response in both diseases, which also shares similarities to complement activation during BM (Molins et al., 2018).

### Pro-inflammatory Responses

Together with complement system activation during BM, the toll-like receptors (TLRs) and NOD-like receptors (NLRs) expressed on or within CNS antigen-presenting cells may become activated by bacterial ligands, triggering the production of various cytokines and chemokines (the “mediators”) that facilitate recruitment of immune cells (the “effectors”) to the central infectious loci to eradicate the bacteria (Mook-Kanamori et al., 2011). At high bacterial infectious doses in the CNS, the “effectors” that also carry the “sensors,” for example, neutrophils expressing TLR2, relentlessly elicit an immune response, tipping the balance of pro-and anti-inflammatory responses towards the former, resulting in an exaggerated immune response and ensuing cytokine storm (Mook-Kanamori et al., 2011, 2012). Similarly, in AD, over-activated microglia contribute to heightened production of inflammatory cytokines, triggering a positive feedback loop; genetic polymorphisms of several cytokines, such as interleukin (IL)-1α, IL-1β, IL-6, and tumor necrosis factor (TNF), modify the risk of AD development and progression in certain populations (Su et al., 2016). In AMD, parainflammation is proposed to be dysregulated when an inciting insult pushes the response beyond a threshold that can be coped with by the normal host autonomous response for cellular repair during aging. This results in excessive activation of resident and recruited immune cells and a subsequent cytokine storm (Chen and Xu, 2015). Since certain immune cells release reactive oxygen species as part of the pathogen-killing process, the excessive production of cytokines and associated infiltration of activated immune cells into affected tissues contributes to heightened oxidative stress. This is postulated to be a synapse/neuron-damaging phenomenon and is observed in both BM and AD/AMD (Bonda et al., 2010; Barichello et al., 2013; Chen and Xu, 2015).

## Discussion

There is growing evidence implicating various microbial infections in the pathogenesis of diseases that historically were not thought to be of infectious origin. A well-known example is gastritis, which was traditionally thought to be caused by stress and other lifestyle factors, but which has become treatable by antibiotic eradication of *Helicobacter pylori* (Abbott, 2005; Ahmed, 2005). Likewise, there is increasing evidence supporting the role of infectious pathogens in the aetiology of cardiovascular diseases (Fong, 2009; Khademi et al., 2019). To add to this list, lines of evidence have emerged that suggest a microbial aetiology for some age-related neurodegenerative diseases. While individual microbial species and phyla have been investigated for their association with AD and AMD, it is noteworthy that the hypothesis of infectious aetiology of neurodegenerative diseases may not require a specific disease-causing microbial strain or variant. The trigger for inflammation that causes damage may be attributable to interactions within the microbial population; for example, dysbiotic oral and gut microbiota have been proposed to play a role in the pathogenesis of AD/AMD (Pritchard et al., 2017; Sochocka et al., 2019; Arjunan, 2020; Floyd and Grant, 2020). It remains unknown whether a keystone pathogen exists, or an infection-initiated/mediated predisposing “immunological signature” contributes to cumulative pathological pathways that ultimately lead to disease development.

### Common Immunopathological Mechanisms

During infection, the complement system is activated to combat intruding pathogens. This represents the first line of defense by host innate immunity during BM (Prasad et al., 2005). Similarly, the complement system is known to play a key role in AD (McGeer and McGeer, 2002; O’Bryant et al., 2010) and AMD (Molins et al., 2018) disease pathogenesis. However, there remains no clear answer about the trigger(s) of complement activation in these diseases. The first theory of AD/AMD aetiology posits the accumulation of endogenous waste products, i.e., amyloid (AD)/drusen (AMD), as the potential trigger. Alternatively, it has been suggested that the presence of infectious agents activates the complement system. BM and AD/AMD share other common inflammatory events (Table 1) that end with pathological inflammation and oxidative stress, resulting in local tissue damage and loss of function. Activated microglia, macrophages, monocytes, and T cells are some of the common cellular immune mediators responsible for disease pathogenesis in BM and AD/AMD, while upregulation of IL-1β and CC chemokine ligand 2 has also been demonstrated in all three diseases (Kaarniranta and Salminen, 2009; Coutinho et al., 2013; Chen and Xu, 2015; Domingues et al., 2017). Furthermore, researchers in the different disease disciplines (BM, AD, or AMD) have separately looked at the role of the proinflammatory cytokine, interferon-gamma (IFN-γ), in disease pathogenesis. It has been proposed that targeting IFN-γ might have therapeutic potential in all three conditions. In our studies of experimental pneumococcal meningitis, we identified an important role of IFN-γ in mediating host immune responses that link to enduring neurological impairments in mice that survived following antibiotic treatment (Too et al., 2014). We found that IFN-γ-deficient mice treated with the antibiotic ceftriaxone survived pneumococcal meningitis with reduced cognitive and behavioral disorders compared to their wild-type counterparts and that the nexus between the toll-like receptors (TLRs) 2 and 4, IFN-γ and the enzyme indoleamine 2,3-dioxygenase-1 contributed, at least in part, to the neurological sequelae resulting from pneumococcal meningitis (Too et al., 2014; Too and Mitchell, 2021). Similarly, neutralising IFN-γ in a transgenic mouse model of AD was found to ameliorate behavioral deficits and amyloid plaque burden (Browne et al., 2013). In the context of AMD, analysis of serum IFN-γ in AMD patients has given mixed results (Afarid et al., 2019; Litwinska et al., 2019). Interestingly, though, in human RPE-derived ARPE-19 cells, recent evidence suggests that this cytokine induces expression of BRAF-activated non-coding RNA (BANCR), a regulatory transcript involved in immunopathological processes (Kutty et al., 2018); inhibition of IFN-γ has therefore been postulated as a potential therapeutic option in AMD.

### Lessons Learned From BM

Despite there being no direct serological evidence that links causative pathogens of BM to AD/AMD, a decreased cerebrospinal fluid level of Aβ42—a biological phenomenon of AD—was found in patients with acute purulent BM (Sjogren et al., 2001). Moreover, the disease-causing immunopathological pathways seen during the acute phase of BM partially resemble the active disease stage of AD/AMD when host inflammatory responses tip towards detrimental effects (Table 1). While correlations between BM and age-related diseases have rarely been investigated, it is worth mentioning that the lifelong cognitive and behavioral disorders among young BM survivors may predispose them to reduced cognitive reserve during later life, which increases the risk of AD (Stern, 2012). Furthermore, in the context of BM, although antibiotic treatment substantially reduces the mortality rate, survivors often exhibit neurological disorders upon recovery. The process of bacterial clearance by antibiotics, and bacterial autolysis, release immunoactive bacterial components that excessively amplify the host immune response and thereby cause permanent damage to host tissue (Brouwer et al., 2010). In this regard, effective treatment for BM can conceivably be achieved by optimally suppressing key pathological immune processes without compromising bacterial clearance. The search for an adjunctive treatment that mitigates the augmented inflammatory response, to be administered alongside a non-bacteriolytic antibiotic, may be a promising therapeutic direction (Bewersdorf et al., 2018).

In light of these data, two fresh insights can be taken from BM research. First, an immunomodulatory approach may not work to ameliorate AD/AMD if microbial agents remain in the host system (e.g., in the case of dysbiotic microbiota), since it may affect the host’s innate immunity to combat pathogens. Second, broad-spectrum antibiotic treatment may not be feasible if the source of inflammation is unknown. For instance, attempting treatment with a bacteriolytic antibiotic in AD/AMD patients who may have been infected with pathogens that are prone to release immunoactive components when lysed, might well contribute to an undesirable outcome. Moreover, the administration of broad-spectrum antibiotics may unfavorably alter the oral/gut microbiome. Several clinical trials have tested the efficacy of antibiotics in preventing or ameliorating cognitive impairments in AD. The findings remain inconsistent. For instance, antibiotic treatment with doxycycline and rifampin of patients with mild to moderate AD, whose medical history of infections was unclear, demonstrated protection against cognitive decline in a 2004 clinical trial (Loeb et al., 2004), but not in a 2013 trial (Molloy et al., 2013). Not identifying the source of inflammation may have contributed to the inconsistent findings seen in these clinical trials (Loeb et al., 2004; Molloy et al., 2013). An immunomodulatory therapy as an adjunct to antibiotic treatment may be useful to alleviate inflammation-driven catastrophic events without compromising host-pathogen clearance.

### Open Questions

Our understanding of any parainfectious aetiology of age-related neurodegenerative diseases remains in its infancy. The mechanisms of AD and AMD are multifactorial, as a consequence of the range of phenotypes and the stereotypical nature of pathological responses to disease. These conditions may each represent a final common pathway to a variety of disease-causing processes which are clinically grouped as single entities. To advance AD and AMD research, we propose that several issues will need to be addressed:

1. Multiple microbes are serologically, histopathologically, or molecularly associated with AD or AMD, but none of them have been causatively related to either disease by fulfilling Koch’s postulates for the establishment of a causative link between a microbe and a disease (Tabrah, 2011). It, therefore, remains a question whether the different microbes can independently cause the disease, or a dysbiotic microbiome is responsible for the disease development. If the latter is true, it predicates caution in further clinical trials to treat AD with antibiotics, since such therapy might cause unfavorable alterations in the microbiome.
2. Given the existing evidence that supports an infectious aetiology of AD and AMD, we will need to explore when, where, and how the microbes initiate the pathological mechanisms.
3. Findings from microbiological and immunopathological research have led us to understand that numerous infectious diseases induce some common immunopathological pathways, such as classic complement pathway activation, cytokine storm, and oxidative stress. It is important, therefore, to identify any disease-specific immunopathological factor(s), to facilitate the exploitation of therapeutic targets. In light of findings from BM research that has explored therapies to prevent infection-associated neurological disorders, a potential treatment to block AD and AMD development or progression might potentially combine antibiotics with immunomodulatory agents.

## Author Contributions

LKT wrote the manuscript. NH and MPS revised the manuscript. All authors contributed to the article and approved the submitted version.

## Conflict of Interest

The authors declare that the research was conducted in the absence of any commercial or financial relationships that could be construed as a potential conflict of interest.
